# Comprehensive plant health monitoring: expert-level assessment with spatio-temporal image data

**DOI:** 10.3389/fpls.2025.1511651

**Published:** 2025-05-30

**Authors:** Alvaro Fuentes, Syed Ali Asgher, Jiuqing Dong, Yongchae Jeong, Mun Haeng Lee, Taehyun Kim, Sook Yoon, Dong Sun Park

**Affiliations:** ^1^ Department of Electronics Engineering, Jeonbuk National University, Jeonju, Republic of Korea; ^2^ Core Research Institute of Intelligent Robots, Jeonbuk National University, Jeonju, Republic of Korea; ^3^ ³Department of Smart Farm, Chungnam State University, Chungcheongnam, Republic of Korea; ^4^ Department of Agricultural Engineering, National Institute of Agricultural Sciences, Wanju, Republic of Korea; ^5^ Department of Computer Engineering, Mokpo National University, Muan, Republic of Korea

**Keywords:** plant health assessment, deep learning, spatiotemporal imaging, precision agriculture, tomato phenotyping

## Abstract

Maintaining crop health is essential for global food security, yet traditional plant monitoring methods based on manual inspection are labor-intensive and often inadequate for early detection of stressors and diseases, and insufficient for timely, proactive interventions. To address this challenge, we propose a deep learning-based framework for expert-level, spatiotemporal plant health assessment using sequential RGB images. Our method categorizes plant health into five levels, ranging from very poor to optimal, based on visual and morphological indicators observed throughout the cultivation cycle. To validate the approach, we collected a custom dataset of 12,119 annotated images from 200 tomato plants across three varieties, grown in semi-open greenhouses over multiple cultivation seasons within one year. The framework leverages state-of-the-art CNN and transformer architectures to produce accurate, stage-specific health predictions. These predictions closely align with expert annotations, demonstrating the model’s reliability in tracking plant health progression. In addition, the system enables the generation of dynamic cultivation maps for continuous monitoring and early intervention, supporting data-driven crop management. Overall, the results highlight the potential of this framework to advance precision agriculture through scalable, automated plant health monitoring, guided by an understanding of key visual indicators and stressors affecting crop health throughout the cultivation period.

## 1 Introduction

Agricultural productivity is increasingly threatened by pathogens, pests, and environmental stressors, making global food security a growing challenge (Gai and Wang, 2024). According to United Nations (UN) population projections, the global population is expected to reach 9.7 billion by 2050, further increasing the demand for agricultural output (Domingues et al., 2022). However, the agricultural sector faces significant challenges, including fungal and bacterial diseases, extreme weather conditions, and shifting soil properties. These factors collectively contribute to annual economic losses exceeding 220 billion USD (Singh et al., 2019). Addressing these challenges requires innovative solutions for proactive crop health monitoring and effective management strategies to sustain agricultural productivity and food security (Singh et al., 2023).

Traditional plant health assessment methods, such as manual visual inspections by farmers or agricultural experts, remain widely used (Ghazal et al., 2024). While these approaches rely on expertise and experience, they are labor-intensive, prone to human error, and impractical for large-scale or remote farms. Furthermore, variations in crop species, evolving disease patterns, and the emergence of new pathogens often lead to misdiagnosis or delayed interventions (Kong and Yang, 2023). Consequently, there is an urgent need for automated, accurate, and scalable plant health monitoring systems that provide farmers with actionable insights (Aijaz et al., 2025).

Recent advancements in deep learning (DL) have significantly improved plant disease detection by leveraging image-based techniques (Sajitha et al., 2024). Convolutional neural networks (CNNs) have played a pivotal role as feature extractors, demonstrating remarkable accuracy in identifying plant diseases (Ferentinos, 2018). For instance, Mohanty et al. (2016) achieved a breakthrough in automated plant disease detection by classifying 26 diseases across 14 crops using CNN models. Argüeso et al. (2020) further optimized this process by employing few-shot learning with Siamese networks and Triplet loss, reducing training data requirements by 90%. Similarly, Deng et al. (2021) applied ensemble learning with ResNeSt-50, SE-ResNet-50, and gDenseNet-121 to detect six rice diseases, effectively lowering misdiagnosis rates. More recently, Kalpana et al. (2024) introduced an ensemble model combining Swin transformers and residual convolutional networks, demonstrating improved performance on the Plant Village dataset. Additionally, recent research has explored solutions for low-data scenarios, such as few-shot learning (Mu et al., 2024; Rezaei et al., 2024) and contrastive learning for pre-training and fine-tuning on small labeled datasets (Zhao et al., 2023).

Beyond plant disease classification, researchers have addressed dataset limitations by focusing on localized symptom detection. Object detection and segmentation models have been integrated with CNNs to identify specific regions of interest, enabling the detection of multiple symptoms within the same image. Fuentes et al. (2017, 2018, 2021b) applied object detection algorithms to recognize tomato diseases and pests using a custom dataset of tomato plant diseases. Roy et al. (2022) enhanced the YOLOv4 framework for tomato disease identification by incorporating DenseNet and additional residual blocks, achieving 96.29% accuracy on 1,200 images from the Plant Village dataset. Similarly, Alqahtani et al. (2023) introduced PlantRefineDet, a method that utilizes ResNet-50 as a feature extractor with RefineDet to recognize crop disorders across 38 category groups.

Further studies have explored adapting these models to real-world conditions, tackling challenges such as domain shift (Fuentes et al., 2021a), unknown and out-of-distribution disease recognition (Meng et al., 2023; Dong et al., 2024a), data availability constraints (Xu et al., 2022), and cross-crop plant disease recognition using visual-language and iterative learning-guided models (Dong et al., 2024b). These ongoing efforts continue to refine plant disease detection systems, enhancing their robustness and applicability in practical agricultural settings.

Despite these advancements, we identify several remaining challenges in plant health assessment:

1. Data Collection – Many plant disease recognition frameworks rely on datasets collected under controlled conditions, focusing on specific diseases or crop types. These datasets may not fully represent real-world agricultural variability, including differences in lighting, plant growth stages, or environmental stressors (Kendler et al., 2022). Expanding datasets with diverse and high-quality samples and labels is crucial for improving model generalization and enhancing model robustness across different crops and field conditions (Dong et al., 2022, 2023b, 2023a).
2. Sequential Plant Health Assessment – Most existing models perform single-instance disease classification (Salman et al., 2023), lacking the capability to track plant health over time. Continuous monitoring and time-series analysis could enhance early detection, enabling proactive interventions before symptoms become severe (Javidan et al., 2024). This approach also offers a deeper understanding of plant growth progression and overall health status.
3. Domain Shift – Models trained on specific datasets often struggle when deployed in different agricultural environments due to variations in imaging devices, climate conditions, soil properties, and crop physiology (Xu et al., 2023). Addressing domain shift requires techniques such as domain adaptation (Busto and Gall, 2017), transfer learning, and continual learning to ensure model robustness in diverse settings.

By addressing these challenges, AI-driven plant health assessment can evolve beyond theoretical accuracy to become a reliable and adaptable tool for modern agriculture. This involves understanding plant health indicators throughout the entire cultivation cycle. Such comprehensive monitoring is essential for effective crop management, allowing for the timely identification of changes and implementing prompt interventions when anomalies arise. Consequently, this approach can help prevent losses, identify underlying causes, and design control strategies tailored to specific crop varieties and growth stages—an essential aspect of controlled environment agriculture.

To address these issues, this study presents a spatio-temporal plant health monitoring framework that leverages image-based deep learning techniques to assess plant health throughout the entire cultivation period. Unlike traditional disease detection models, our framework analyzes plant health-related features continuously, categorizing plant health into five levels—from very poor to optimal—based on a wide range of visual and morphological indicators. To validate the effectiveness of this approach, we developed a custom dataset comprising over 12,000 high-resolution images of individual tomato plants captured in semi-open greenhouse environments. This dataset, which includes multiple growth stages and tomato varieties cultivated across various seasons, represents a significant contribution to agricultural AI research.

**Figure 1:**
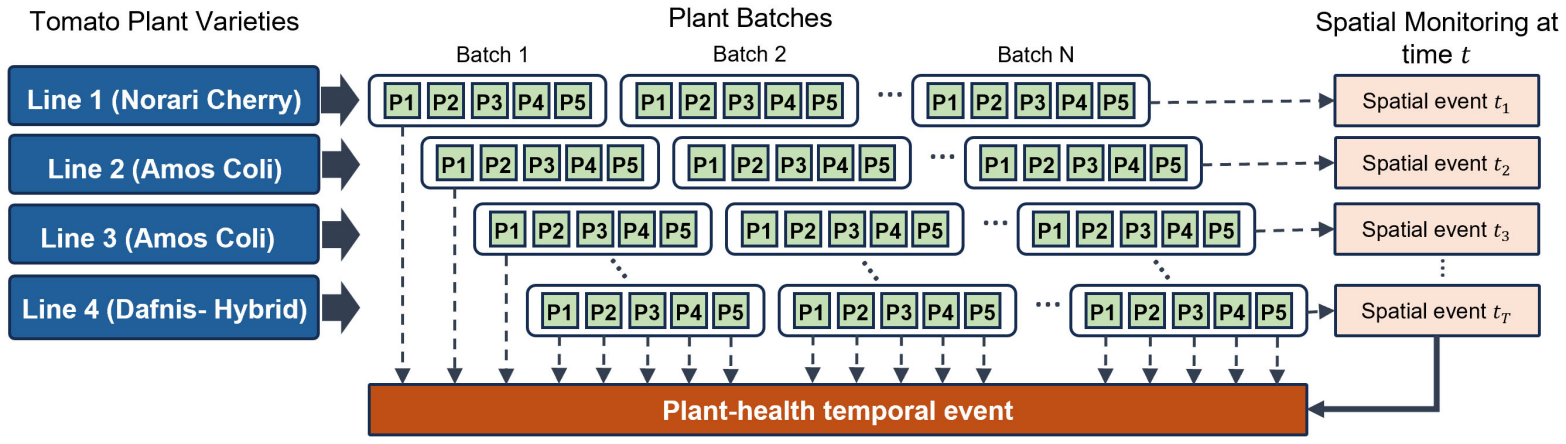
Spatio-temporal plant health assessment strategy. This diagram represents the research objective of continuously monitoring plant health throughout the entire cultivation period, enabling precise assessment and tracking of health status over time.

The proposed framework centers on the sequential assessment of plant health, offering accurate evaluations at distinct growth stages. This approach facilitates the creation of cultivation process maps that monitor plant health progression over time, supporting data-driven decision-making and timely interventions by farmers. **Figure 1** presents the strategic objective of this research—continuous monitoring of individual plants throughout the entire cultivation cycle. The study utilizes data collected from four cultivation lines, aiming to assess plant conditions at specific spatial points. By aggregating these observations across time, we construct temporal health profiles that capture dynamic changes in plant status over the full growing period.

The key contributions of this study are described below:

1. Spatio-Temporal Tomato Plant Dataset – We collected and labeled a dataset of approximately 12,000 images representing various tomato varieties (including cherry tomatoes, large tomatoes, and the Dafnis hybrid) across two cultivation periods lasting approximately one year. The dataset focuses on monitoring a total of 200 individual plants, with images captured weekly from the plant’s growth point (top section) throughout the cultivation cycle. This dataset is unique, as no existing dataset provides similar characteristics.
2. Data Annotation Strategy –We propose a five-point annotation scale (1–5) to assess plant health, ranging from poor to optimal. As part of the comprehensive framework, a domain expert guided the temporal labeling process, incorporating phenotypic indicators such as stem thickness, leaf condition, and overall plant vitality to ensure accuracy.
3. Framework for Plant Health Monitoring –We introduce an image-based deep learning framework that categorizes plant health into five distinct levels using state-of-the-art feature extractors. This approach enables a comprehensive assessment of plant health across the entire cultivation period, facilitating long-term tracking of individual plant health.

The remainder of this paper is structured as follows: Section 2 describes the dataset acquisition, annotation strategy, and proposed methodology. Section 3 presents the implementation details and experimental results. Section 4 discusses the limitations and strengths of this research. Section 5 concludes the paper by summarizing the findings and outlining future research directions.

## 2 Materials and methods

### 2.1 Dataset acquisition

This study was conducted at the Fruit Vegetable Research Institute in Buyeo, South Korea, using a collected dataset of high-resolution RGB images of three tomato plant varieties: Amos Coli, Nonari-Cherry Tomato, and Dafnis-Hybrid. These varieties were cultivated under standard grower-managed conditions in semi-open greenhouses over two consecutive periods: January–July and August–December 2022. **Table 1** details the plant varieties and cultivation periods.

**Table 1:** Data acquisition details for tomato plant health monitoring.

Cultivation Line (within the greenhouse)	Tomato Plant Variety	Cultivation Period	Number of Weeks	Number of Plants
1	Nonari-Cherry	2022/08 – 2022/12	18	50
2	Amos Coli	2022/01 – 2022/07	25	50
3	Amos Coli	2022/01 – 2022/07	25	50
4	Dafnis-Hybrid	2022/08 – 2022/12	18	50

“Line” refers to the cultivation line in the greenhouse where the plants were grown.

During the first 25-week period, 100 Amos Coli plants were monitored in Crop Lines 2 and 3 of the greenhouse. In the second 18-week period (August–December), 100 plants, 50 of Nonari-Cherry and 50 of Dafnis-Hybrid were observed. In total, 200 plants were monitored over one year. Weekly site visits were conducted throughout the cultivation periods to capture high-resolution RGB images. Three images were taken per plant from different viewpoints—left, right, and top—focusing on the upper plant region, a key indicator of growth (Cho et al., 2023). This multi-view strategy enabled a comprehensive assessment of plant health.

**Figure 2:**
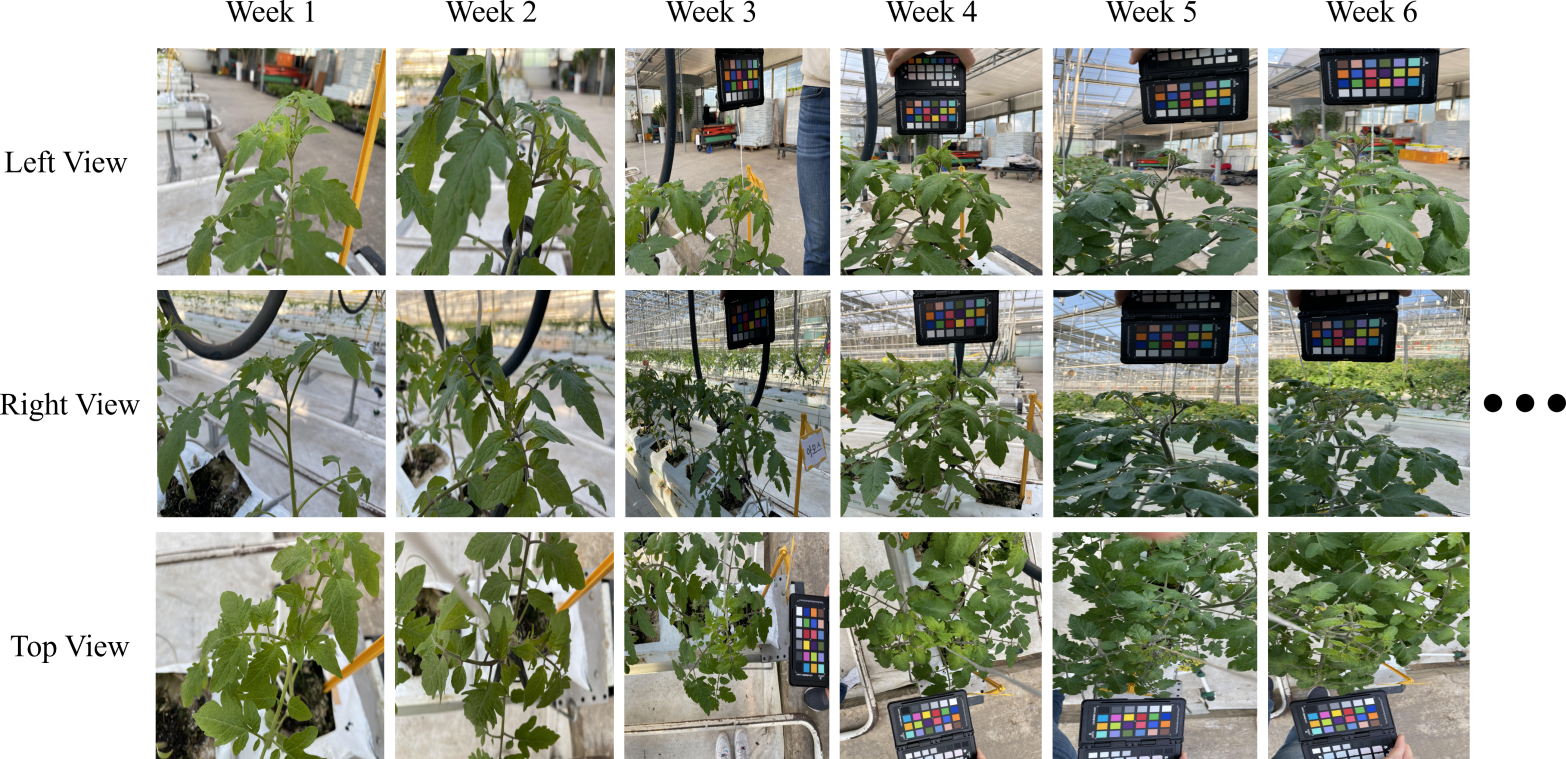
Sample images from three viewpoints showing the growth progression of a plant over multiple weeks.

**Figure 2** presents sample images from various viewpoints collected for several weeks. Images were captured using smartphone cameras, with a color checker included for future color-based leaf analysis. Each plant was tagged with a QR code containing its plant number, slab number, and cultivation period for identification.

### 2.2 Dataset Annotation by a domain expert

**Figure 3:**
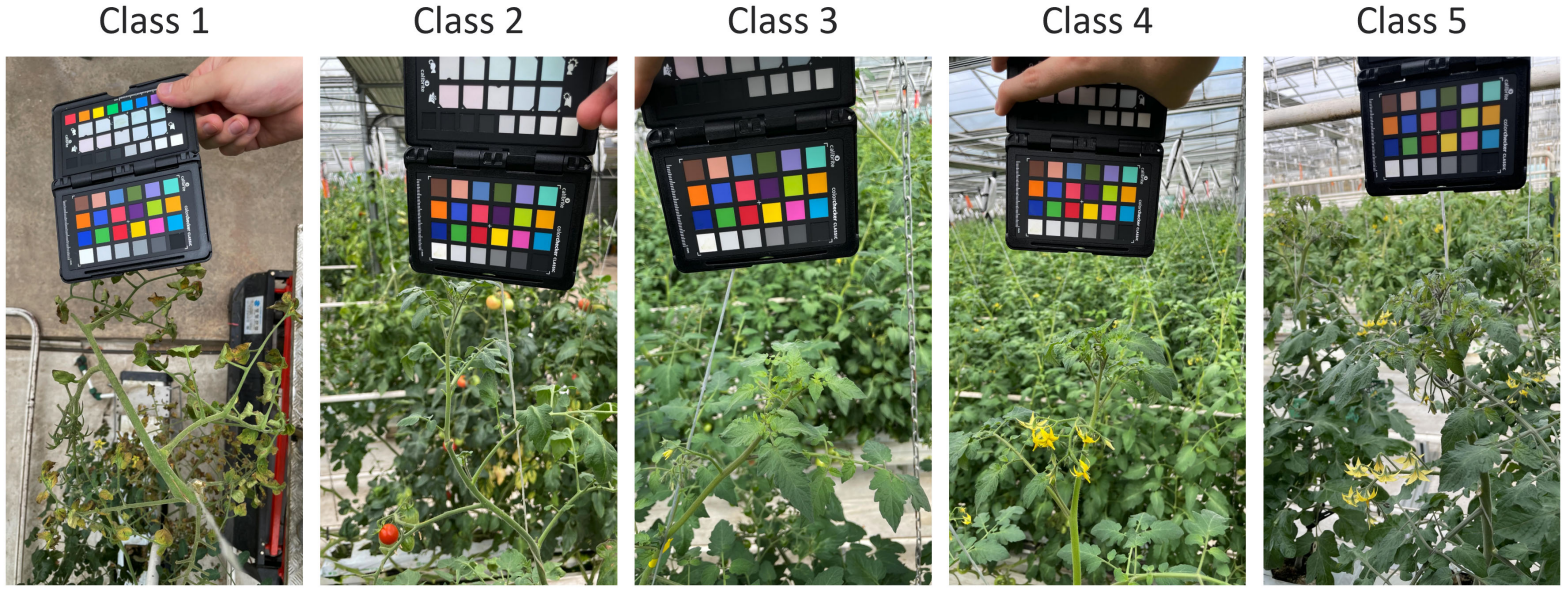
Example images illustrating the five health status categories, from class 1 (severe health deterioration) to class 5 (optimal health), providing visual references for the rating strategy used in annotation.

Following data collection, a domain expert in plant physiology from the Fruit Vegetable Research Institute in Buyeo, South Korea, meticulously annotated each image in the dataset, documenting various growth stages and conditions of tomato plants. Each image was assigned a health status level on a scale from 1 to 5. **Table 2** provides detailed descriptions and specific indicators used for classification, while **Figure 3** presents representative images for each health level, ranging from Class 1 (severe health issues) to Class 5 (optimal health), serving as visual references for the annotation process.

**Table 2:** Health status level, categories, and indicators used by the domain expert to assess plant health.

Health Status Level	Category	Diagnostic Indicators
5	Optimal health	Vibrant green leaves, uniform growth, no visible stress, no signs of disease, pests, or deficiencies.
4	Minor deviations from ideal health	Slight discoloration, minor leaf curling or wilting, early-stage nutrient imbalance, small pest presence but no major damage.
3	Moderate health	Noticeable discoloration (yellowing, browning), leaf deformation, moderate pest or disease symptoms, stunted growth, and some necrotic spots.
2	Poor health	Extensive discoloration, widespread necrosis, severe pest infestation, stunted growth, and significant wilting or leaf drop.
1	Very poor health	Severe leaf damage or defoliation, major necrosis, severe stunting, significant pest or disease damage, plant near death.

During labeling, we identified cases of plant health deterioration and their potential causes. While most plants remained in optimal condition, occasional issues arose, such as valve malfunctions that disrupted nutrient supply. In some instances, this led to sudden plant distress and, in severe cases, plant death. However, when problems were detected early, growers were able to intervene and correct the issue, preventing further damage. Further analysis of plant growth and health progression is presented in the Discussion section, supported by representation maps that illustrate the plants’ development over time.

### 2.3 Dataset distribution

**Table 3** presents the distribution of image samples across datasets corresponding to different tomato plant varieties cultivated in Line 1, Line 2, Line 3, and Line 4 within the greenhouse. The number of samples varies significantly across health status ratings. Since the grower’s objective was to maintain optimal plant growth, data acquisition prioritized class levels 4 and 5, which represent plants in near-optimal or optimal conditions. This pattern was consistent across tomato plant varieties, cultivation lines, and seasons.

**Table 3:** Distribution of image samples across cultivation lines.

Datasets (Line)*	Category by Health Status Rating					
	Class 1	Class 2	Class 3	Class 4	Class 5	Total
Line 1	18	27	756	1,412	341	2,554
Line 2	9	21	324	1,743	1,509	3,606
Line 3	30	450	933	1,266	889	3,568
Line 4	27	22	73	504	1,738	2,364
Total	84	520	2,086	4,952	4,477	12,119

* The corresponding plant variety is listed in **Table 1**.

In contrast, although less frequently, data was also collected for class levels 3 to 1, representing cases where plant health deteriorated during the monitored period. These lower health levels provide insights into plant stress factors and potential causes of deterioration. After annotation by domain experts, the dataset was structured based on the assigned health status for each image, ensuring a well-balanced representation of plant conditions throughout the study. We will refer to each dataset as the corresponding line and number to facilitate the description.

### 2.4 Proposed plant health monitoring framework

**Figure 4:**
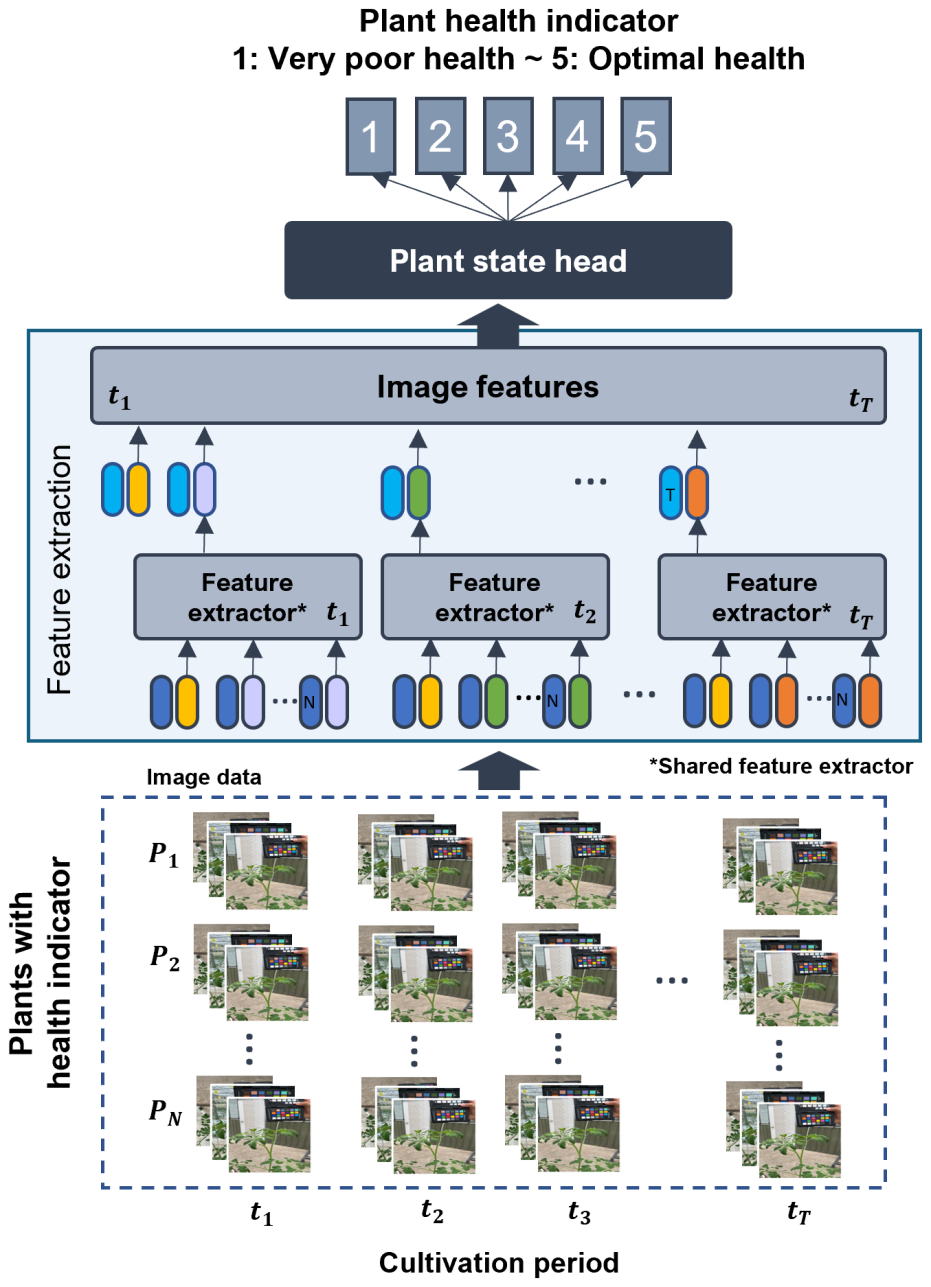
Schematic representation of the image-based plant health monitoring framework. The input consists of plant images collected throughout the entire cultivation period, while the output is a plant health state indicator that determines the plant’s condition at a specific time (*t*). P# represents plant identification numbers, and t# denotes specific time points for health monitoring. * represents “shared feature extractor”.

Our deep learning-based plant health monitoring framework (**Figure 4**) consists of multiple stages, beginning with the collection of high-resolution RGB images from semi-open greenhouse environments throughout the cultivation period. These images were annotated by domain experts, assigning health status labels ranging from 1 (very poor health) to 5 (optimal health). These labels serve as the ground truth for training deep learning models. A comparative evaluation was performed using a separate validation set to identify the most accurate and reliable model for plant health monitoring. Various techniques were applied to enhance dataset diversity and robustness, as detailed in the implementation section.

#### 2.4.1 Feature extraction and model architectures

At the core of our framework is a deep learning-based image classification framework, leveraging state-of-the-art neural network architectures to assess plant health automatically. The proposed framework integrates CNN-based and transformer-based architectures. Each applied network uses all the settings provided in the original architectures in terms of layer configuration. The distinguishing changes relate mainly to finding the proper parameters for the applications. Specifically, to develop our approach, we leverage the following architectures:

1. CNN-based Models – The feature extraction mechanism in CNN models involves a sequential arrangement of convolutional layers, followed by pooling layers, and finally, at the end, fully connected layers with SoftMax activation for classification (Rangel et al., 2024).
  - ResNet-18 (He et al., 2015): Introduces deep residual learning, addressing the vanishing gradient problem commonly found in deep networks. It consists of 18 layers, structured into residual blocks, where shortcut (identity) connections allow information to bypass certain layers. Each residual block consists of two to three convolutional layers, followed by batch normalization and ReLU activation. ResNet-18 is computationally lighter than deeper ResNet variants, making it well-suited for real-time agricultural applications where efficiency is critical.
  - VGG-16 (Simonyan and Zisserman, 2014): Consists of 16 layers, including 13 convolutional layers and three fully connected layers. It uses small 3 × 3 convolutional kernels, allowing deeper feature extraction while maintaining computational efficiency. This architecture employs ReLU activation and max-pooling layers to downsample feature maps progressively. VGG-16 has been widely adopted for image recognition tasks, including plant disease detection, due to its ability to learn fine-grained texture details, which are crucial for distinguishing plant health conditions.
  - ConvNeXt (Liu et al., 2022): A modern CNN combining group convolutions, inverted bottleneck structures to improve feature extraction and reduce computational load. This network incorporates group convolutions and an inverted bottleneck structure to improve feature extraction and reduce computational load. Advanced techniques like AdamW optimizer (Loshchilov and Hutter, 2017), Mixup (Zhang and Cisse, 2018), Cutmix (Yun et al., 2019), RandAugment (Cubuk et al., 2020), Stochastic Depth, and Label Smoothing (Müller et al., 2019) further optimize its performance.
2. Transformer-based Models – Unlike CNNs, transformer-based architectures do not rely on spatial hierarchies but instead use self-attention mechanisms to model both local and global dependencies within images (Vaswani et al., 2017). These models have demonstrated superior performance in vision tasks, including plant health assessment.
  - Vision Transformers – ViT (Dosovitskiy et al., 2020): Processes images by dividing them into fixed-size patches that are linearly embedded with positional encoding. In this network, patch embeddings pass through multi-head self-attention layers, capturing complex spatial dependencies across the image. This study employs the ViT Base model, which consists of 12 transformer layers, a hidden size of 768, and a 16 × 16 patch size.
  - Swin Transformers (Liu et al., 2021): Enhances ViTs by partitioning images into non-overlapping local windows and computing self-attention within these regions, significantly reducing computational cost. The shifted windowing mechanism allows for cross-window interactions, improving spatial feature learning at multiple scales. This study utilizes the Swin Transformer Base model, configured with a 4 × 4 patch size and a 7 × 7 local window size to effectively balance accuracy and efficiency.

Based on the feature extraction and plant state classification process shown in **Figure 4**, we define an equation (Equation 1) that mathematically represents the contribution of the approach in predicting the plant state over time.


(1) $$S_{t}=f(\sum_{i=1}^{N}\emptyset (F_{t_{i}}))$$

where $S_{t}$ represents the predicted plant state at time *t*, categorized into states 1 to 5; $F_{t_{i}}$ represents the image features extracted at time *t* for an $i^{th}$ input image; $\emptyset (F_{t_{i}})$ is the feature transformation function that processes the extracted features for representation; and f(.) represents the plant state head, which generates the final plant state according to the established indicators.

By utilizing flexible CNN-based and transformer-based architectures for feature extraction, the proposed method ensures a robust and scalable plant health assessment, optimizing classification accuracy while maintaining computational efficiency.

#### 2.4.2 Performance metrics

To assess the effectiveness of our image-based plant health monitoring framework, we compute the following key performance metrics: accuracy (Equation 2), precision (Equation 3), recall (Equation 4), and F1-score (Equation 5). These metrics provide a comprehensive evaluation of the model’s classification performance.


(2) $$Accuracy=\;\frac{TP+TN}{TP+FP+FN+TN}$$

(3) $$Precision=\frac{TP}{TP+FP}$$

(4) $$Recall=\frac{TP}{TP+FN}$$

(5) $$F1-score=2\times \frac{precision\times recall}{precision+recall}$$

where TP represents True Positive, TN represents True Negative, FP represents False Positive, and FN represents False Negative.

## 3 Experimental results

### 3.1 Implementation settings

The proposed framework was implemented using Python 3.8 and the PyTorch deep learning library (version 1.10.1) with CUDA 11.3 for GPU acceleration. All experiments were conducted on a server equipped with an Nvidia GeForce RTX 3090 GPU, featuring 24,268 MB of memory to efficiently handle deep learning computations.

#### 3.1.1 Data preprocessing and class imbalance handling

To improve model generalization, a preprocessing step was applied to address class imbalance in the dataset. As shown in **Table 3**, an initial inspection revealed significant disparities in sample distribution across classes. For instance, in Line 1, Classes 1 and 2 had only 18 and 27 samples, respectively, whereas Classes 3, 4, and 5 contained 756, 1,412, and 341 samples, respectively. A similar imbalance was observed in Lines 2, 3, and 4, where lower-class samples were underrepresented. To mitigate this issue, classes with fewer than 30 samples (representing fewer than 10 plants) were excluded. The remaining data was then randomly split into training and validation sets, maintaining an 80:20 ratio—with 80% allocated for training and 20% for validation, as detailed in **Table 4**.

**Table 4:** Distribution of training and validation sets per dataset representing plant varieties.

Datasets	Training Data	Validation Data
Line 1	2007	502
Line 2	2860	715
Line 3	2838	709
Line4	1852	463

#### 3.1.2 Model training and optimization

The classification models described in Section 2.4 were applied using transfer learning, leveraging ImageNet pre-trained weights for fine-tuning. To adapt models to our dataset, the last fully connected layers were modified to match the target dataset’s output classes corresponding to the plant health indicator. All pre-trained layers remained trainable, ensuring adaptation to our specific problem. The final activation function was SoftMax, and categorical cross-entropy loss was used as the loss function.

To optimize training efficiency, learning rate schedulers were incorporated. Specifically, we utilized the AdamW optimizer (Loshchilov and Hutter, 2017), initialized with a learning rate of 3e-6, and weight decay of 8e-2. Additionally, a custom learning rate scheduler was implemented, combining warmup and cosine annealing techniques. The learning rate was gradually increased during the initial warm-up phase; then, it followed a cosine decay curve, eventually reaching a minimum learning rate of 1e-6. Hyperparameters are outlined in **Table 5**.

**Table 5:** Training and optimization parameters.

Parameter	Method/Value
Optimizer	AdamW
Learning Rate	3e-6 (initial), 1e-6 (minimum)
Weight Decay	8e-2
Learning Rate Scheduler	Warmup + Cosine Annealing
Loss Function	Categorical Cross Entropy
Final Activation	SoftMax

#### 3.1.3 Data augmentation and image normalization

To enhance model robustness, augmentation techniques were applied, including rotation (± 20 degrees), horizontal flipping, random cropping, Cutmix (Yun et al., 2019), Mixup (Zhang and Cisse, 2018) with a smoothing factor of 0.1. Additionally, pixel values were normalized to a standard scale using the mean and standard deviation. Images were then resized to the standard input dimensions of the respective models, 224 × 224 pixels for VGG-16, ResNet-18, and ViT, and 384 × 384 pixels for Swin Transformer. The mini-batch size was set to 32, optimized based on hardware constraints for efficient training.

### 3.2 Quantitative results

This section presents a detailed analysis of our experimental findings across four datasets: Line 1, Line 2, Line 3, and Line 4. We evaluate the performance of various CNN-based and transformer-based deep learning models (Section 2.4) using key performance metrics: accuracy, precision, recall, and F1-score.

Since average accuracy can be misleading in cases of imbalanced datasets (Thölke et al., 2023), we consider F1-score as the primary evaluation metric. The model achieving the highest F1-score is deemed the most effective for plant health assessment. To mitigate overfitting and ensure stable training, early stopping was implemented.

#### 3.2.1 Model performance across datasets

The results, summarized in **Table 6**, indicate that Swin Transformer-B achieved the highest validation accuracy and F1-scores in Line 1 and Line 2, with 83.7% validation accuracy and a 78% F1-score in Line 1, and 81.2% validation accuracy with a 77% F1-score in Line 2. The Swin Transformer’s performance in these datasets suggests that its multi-scale attention mechanism is particularly effective for distinguishing fine-grained plant health conditions, such as detecting early stress symptoms and disease progression. However, while Swin Transformer performed well, its computational cost remains a challenge, making real-time deployment in resource-limited environments difficult.

**Table 6:** Performance metrics across datasets.

Datasets	Deep Learning Architectures	Training Accuracy	Validation Accuracy	Precision	Recall	F1-Score
Line 1 (Norari-Cherry)	VGG-16	0.852	0.809	0.75	0.74	0.75
	ResNet 18	0.866	0.807	0.76	0.71	0.73
	**Swin Transformer-B**	**0.891**	**0.837**	**0.81**	**0.76**	**0.78**
	VIT-B	0.845	0.794	0.77	0.69	0.72
	ConvNeXt-B	0.887	0.813	0.77	0.71	0.74
Line 2 (Amos Coli)	VGG-16	0.760	0.725	0.67	0.55	0.57
	ResNet 18	0.793	0.751	0.72	0.66	0.68
	**Swin Transformer-B**	**0.858**	**0.812**	**0.80**	**0.75**	**0.77**
	VIT-B	0.708	0.6498	0.68	0.66	0.65
	ConvNeXt-B	0.847	0.789	0.77	0.74	0.76
Line 3 (Amos Coli)	VGG-16	0.763	0.743	0.75	0.75	0.75
	ResNet 18	0.829	0.801	0.78	0.77	0.77
	Swin Transformer-B	0.864	0.839	0.81	0.81	0.81
	VIT-B	0.736	0.680	0.67	0.67	0.66
	**ConvNeXt-B**	**0.872**	**0.846**	**0.82**	**0.82**	**0.82**
Line 4 (Dafnis-Hybrid)	VGG-16	0.838	0.794	0.77	0.70	0.73
	ResNet 18	0.875	0.806	0.86	0.67	0.71
	Swin Transformer-B	0.830	0.772	0.79	0.62	0.66
	VIT-B	0.782	0.683	0.64	0.68	0.66
	**ConvNeXt-B**	**0.892**	**0.783**	**0.79**	**0.70**	**0.74**

Values in bold indicate the best-performing model for each crop line.

In contrast, ConvNeXt-B demonstrated superior performance in Line 3 and Line 4, achieving the highest validation accuracy of 84.6% with an F1-score of 82% in Line 3, and 78.3% validation accuracy with an F1-score of 74% in Line 4. ConvNeXt-B provided a competitive balance between classification accuracy and computational efficiency, making it a viable alternative for large-scale agricultural applications. While ConvNeXt-B slightly outperformed Swin Transformer in Line 3, its performance advantage in Line 4, was more pronounced, suggesting that its convolutional-based structure generalizes better in datasets with more variability in plant health conditions.

#### 3.2.2 Class wise performance analysis

A class-wise breakdown of precision, recall, and F1-score for the best-performing models in each dataset is provided in **Table 7**. The results highlight significant disparities in model effectiveness across different plant health states.

- Class 5 in Line 3 exhibited the highest performance, with a precision of 0.90 and an F1-score of 0.89, likely due to the availability of a sufficient number of training samples.
- Class 2 in Line 3 recorded the lowest F1-score (0.74), reinforcing the impact of data imbalance on classification accuracy.
- Class 5 in Line 1 had an F1-score of 0.64, significantly lower than other classes, demonstrating the impact of sample imbalance on classification performance.
- Swin Transformer excelled in Class 3 (F1-score = 0.79) and Class 4 (F1-score = 0.86) in Line 1, but underperformed in Class 5, indicating that certain plant health conditions may be harder to distinguish without additional contextual features.

**Table 7:** Class-wise performance metrics across datasets.

Datasets	Classes	Precision	Recall	F1-Score
Line 1 (Norari-Cherry)	Class 3	0.90	0.84	0.79
	Class 4	0.81	0.88	0.86
	Class 5	0.70	0.59	0.64
Line 2 (Amos Coli)	Class 3	0.79	0.62	0.69
	Class 4	0.80	0.80	0.80
	Class 5	0.80	0.84	0.82
Line 3 (Amos Coli)	Class 2	0.73	0.75	0.74
	Class 3	0.82	0.76	0.79
	Class 4	0.84	0.89	0.86
	Class 5	0.90	0.88	0.89
Line 4 (Dafnis-Hybrid)	Class 3	0.86	0.55	0.67
	Class 4	0.75	0.79	0.77
	Class 5	0.77	0.77	0.77

These findings underscore the importance of dataset-balancing strategies to mitigate performance degradation in underrepresented classes. Therefore, further improvements may include adjusted sampling techniques, cost-sensitive learning methods, or hybrid architectures that incorporate multi-modal data inputs (e.g., combining RGB images with environmental sensor data).

### 3.3 Confusion matrixes

**Figure 5:**
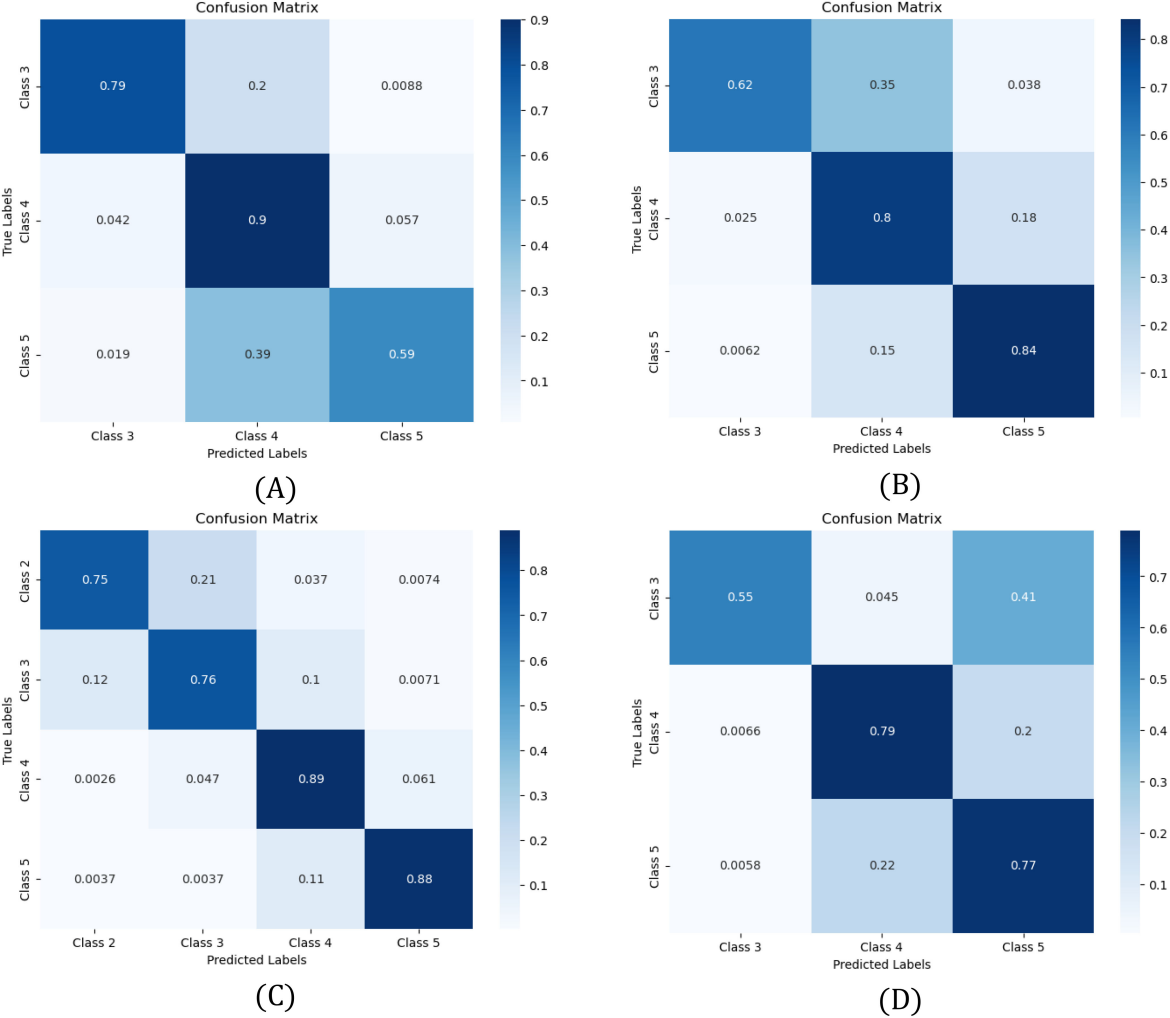
Confusion matrices showing the performance of the model for **(A)** Line 1, **(B)** Line 2, **(C)** Line 3, and **(D)** Line 4. Misclassifications are frequent between adjacent health states due to overlapping characteristics.

To further assess model performance, confusion matrices in **Figure 5** illustrate classification outcomes for each dataset: (A) Line 1, (B) Line 2, (C) Line 3, and (D) Line 4. These matrices highlight correct classifications and misclassifications, offering insights into model strengths and weaknesses. Based on **Table 6**, Swin Transformer was the best model for Lines 1 and 2, while ConvNeXt performed best in Lines 3 and 4.

In Line 2, the model shows strong classification accuracy for Classes 4 (80%) and 5 (84%), but misclassification is frequent between adjacent classes, particularly Classes 3 and 4 (62%). This highlights the challenge of distinguishing gradual health variations, where subtle differences lead to overlaps. A similar trend appears in Line 1, where Class 5 is misclassified as Class 4 in 39% of cases, suggesting the model struggles to differentiate the healthiest plants.

In Line 3 and Line 4, classification follows a similar pattern, with strong diagonal performance but lower accuracy in underrepresented classes. Class 3 in Line 4 and Class 2 in Line 3 exhibit the weakest performance, reinforcing the impact of class imbalance on model reliability. These results emphasize the need for improved data-balancing strategies to enhance the classification of minority health states.

### 3.4 t-SNE visualizations

**Figure 6:**
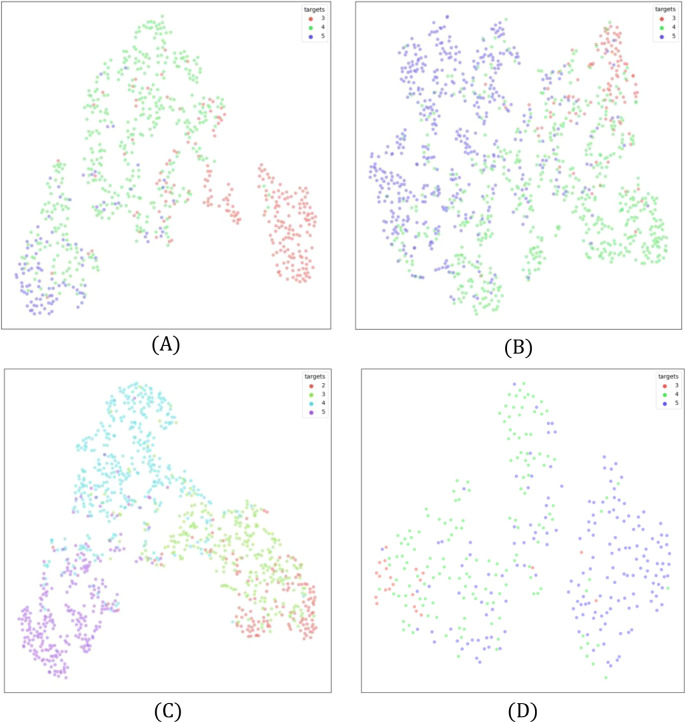
t-SNE plots for **(A)** Line 1, **(B)** Line 2, **(C)** Line 3, and **(D)** Line 4. Overlapping regions indicate classification challenges, particularly in underrepresented classes. Best view in color. Zoom in for better visibility.

The t-SNE plots in **Figure 6** visualize class separability in a reduced two-dimensional space, providing insight into feature distributions. Each point represents a sample, with colors denoting different health states. Overlapping clusters indicate classification challenges, while well-separated clusters suggest effective distinction.

In Line 2 (**Figure 6B**), Class 3 and Class 4 exhibit significant overlap, aligning with confusion matrix findings. Class 3 and Class 5, however, show better separation, reflecting the greater visual difference between moderate and severe health deterioration. Similarly, Line 3 (**Figure 6C**) shows strong overlap between Classes 2 and 3, reinforcing the difficulty in distinguishing early-stage plant stress. In contrast, Class 5 is well separated, confirming distinct features in severely affected plants.

Line 1 and Line 4 follow similar patterns (**Figures 6A, D**), where adjacent health states overlap while distant ones are clearly separated. Notably, better separation is observed in classes with larger training samples, highlighting the role of dataset size in feature learning. These findings stress the importance of augmentation techniques and loss adjustments to improve class differentiation.

### 3.5 Visualization of activation maps

**Figure 7:**
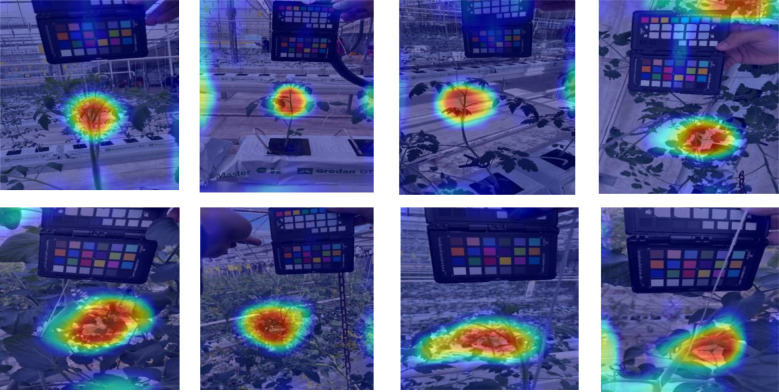
Grad-CAM visualizations for Line 1 and Line 2 datasets. Red regions indicate the most influential areas for classification.

To interpret model decisions, Grad-CAM heatmaps (**Figure 7**) highlight the most influential image regions during classification (Selvaraju et al., 2017). For CNNs, activations were extracted from final convolutional layers, while for transformers, they were taken before the last attention block.

**Figure 8:**
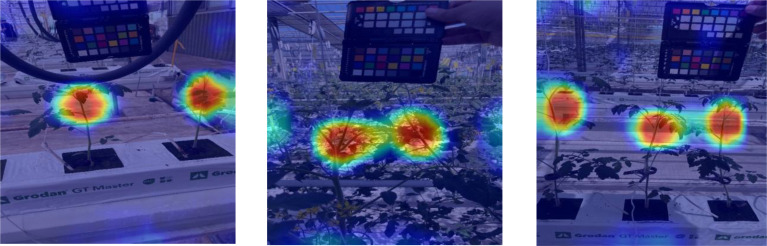
Examples of misclassification due to background plant interference, emphasizing the need for improved spatial focus in model predictions.

Heatmaps show strong activation in the upper plant regions, suggesting that leaf structure, color, and texture play key roles in classification. However, **Figure 8** reveals some challenging cases, especially when models misfocus on background plants, leading to misclassification. This issue arises when target plants are partially occluded or closely positioned to others with different health states. Overall, the trained models capture relevant plant features, yet background interference remains a limitation.

### 3.6 Qualitative results

**Figure 9:**
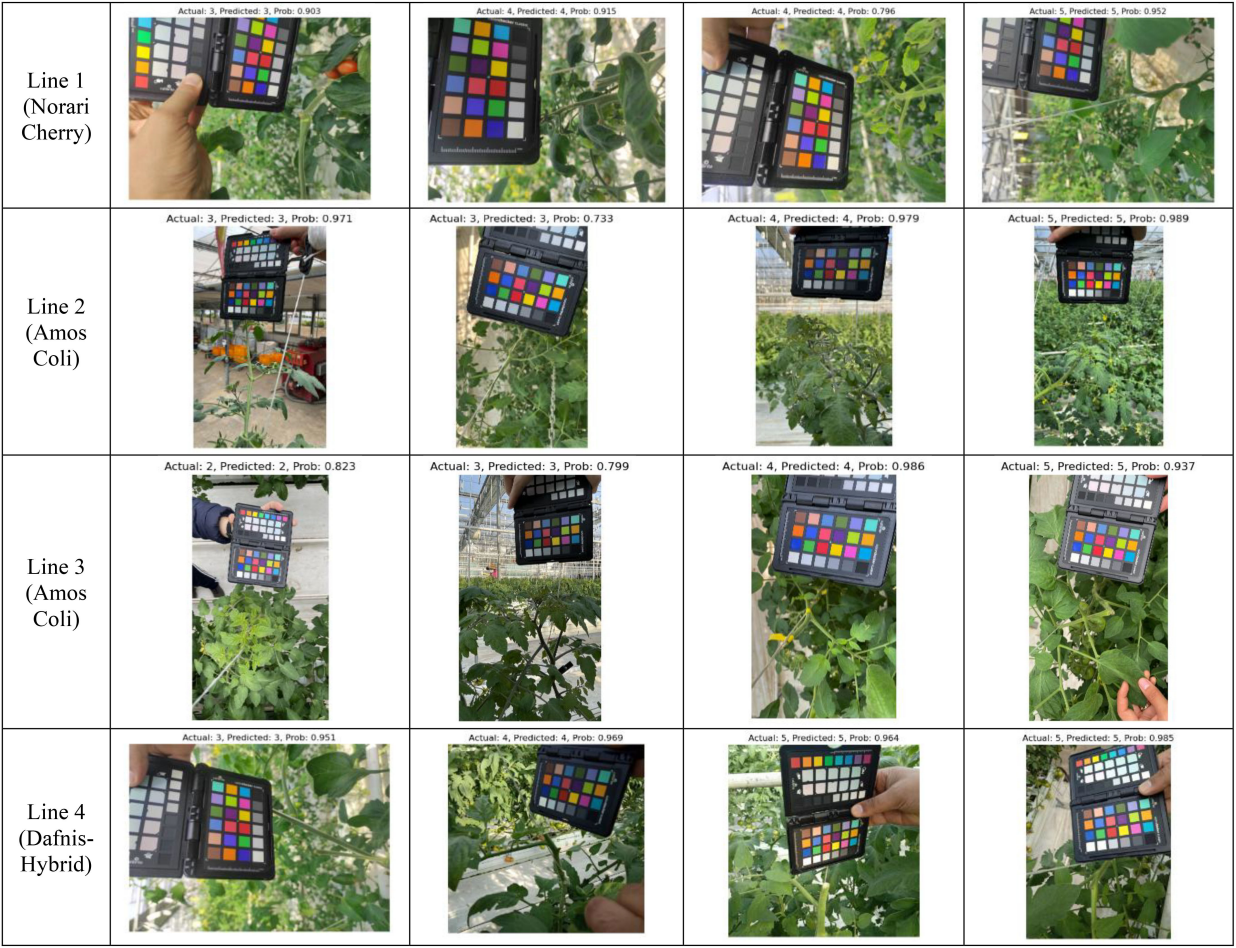
Qualitative evaluation of model predictions across cultivation lines: Line 1, Line 2, Line 3, and Line 4. Each dataset was classified using its top-performing model (Swin Transformer for Lines 1 and 2, ConvNeXt for Lines 3 and 4), demonstrating high-confidence predictions in plant health assessment.

Across all lines, **Figure 9** showcases diverse examples of correctly classified plants, emphasizing the adaptability of each model to its respective dataset. The high-confidence predictions suggest that our models effectively capture key features such as leaf color, shape, and structural integrity, which are indicative of plant health. Moreover, these qualitative results highlight the importance of dataset-specific optimization, as each model exhibits peak performance when applied to the dataset it was best suited for.

Despite these successes, challenges remain in cases where plant health conditions exhibit gradual transitions between classes, which may still contribute to occasional misclassification. This observation aligns with our confusion matrix and t-SNE findings, underscoring the need for further refinements, such as enhanced attention mechanisms and multi-modal inputs integrating environmental factors.

## 4 Discussion

While the proposed framework effectively monitors plant health over time, challenges remain, particularly regarding data availability, distribution, and generalization to new plant samples. To further investigate these limitations, we conducted model validation using plant-based dataset partitioning and spatio-temporal health status modeling. These experiments assess the robustness, adaptability, and long-term applicability of our approach.

### 4.1 Model validation using plant-based dataset partitioning

To evaluate the model’s ability to generalize, we revised the data partitioning strategy by dividing datasets based on individual plants rather than random splits. This setup simulates real-world deployment, where models must classify unseen plants instead of recognizing familiar ones from the training phase.

A major concern with random partitioning is that multiple images of the same plant may appear in both training and validation sets, leading to artificially inflated performance metrics due to memorization rather than generalization. To mitigate this, we allocated 70% of plants for training, 20% for validation, and 10% for testing across all datasets. The best-performing models—Swin Transformer for Line 1 and Line 2, and ConvNeXt for Line 3 and Line 4—were used with the same training strategies described in Section 3.1.

**Table 8** presents the results of this experiment, showing performance comparable to the previous 80:20 random split. These findings confirm that the models retain high classification accuracy even when evaluated on entirely new plants, validating their potential for long-term monitoring and real-world deployment.

**Table 8:** Model performance using plant-based partitioning.

Datasets	Deep Learning Architectures	Training Accuracy	Validation Accuracy	Test Accuracy
Line 1	Swin Transformer-B	0.903	0.804	0.790
Line 2	Swin Transformer-B	0.836	0.797	0.779
Line 3	ConvNeXt-B	0.871	0.827	0.805
Line 4	ConvNeXt-B	0.845	0.774	0.765

### 4.2 Spatio-temporal modeling of plant health over the cultivation period

To comprehensibly understand the generalization of the trained models, we generated spatio-temporal health diagrams using new plants not included in training. These diagrams illustrate the evolution of plant health over time, comparing model predictions with expert annotations.

For each cultivation line, the data of unseen plants was selected, and its health trajectory was predicted using the trained models. These predictions were compared with ground-truth labels provided by domain experts. The models correctly classified health status in 83% of cases, demonstrating strong predictive accuracy for time-series plant health tracking.

**Figure 10:**
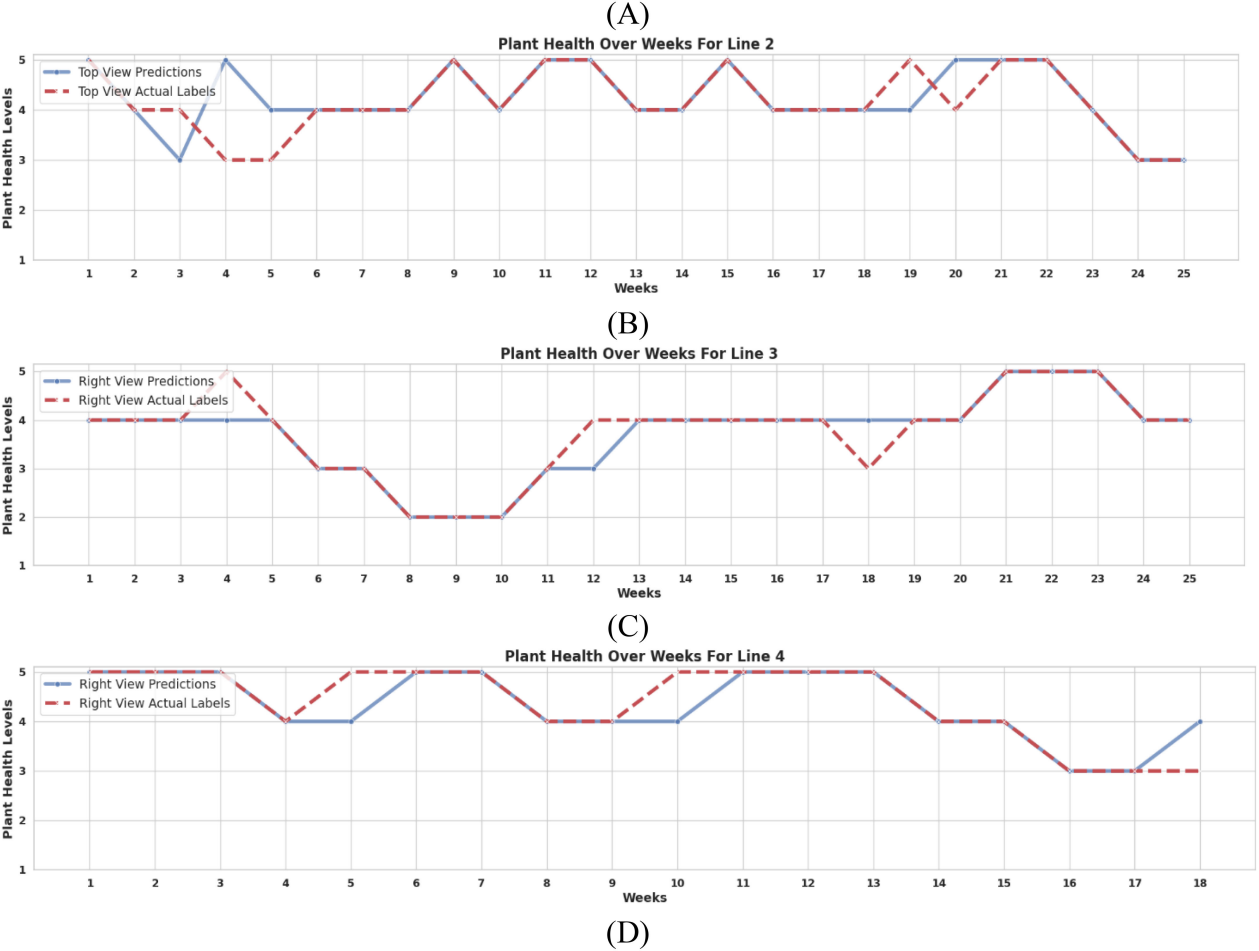
Comparison of model-predicted health status *vs*. expert labels over time for: **(A)** Plant 5 of Line 1, **(B)** Plant 10 of Line 2, **(C)** Plant 1 of Line 3, and **(D)** Plant 21 of Line 4. The dashed red line represents ground-truth health status.

**Figure 10** visualizes health status changes over time, where each subplot represents the progression of an individual plant’s condition. The dashed red line indicates ground truth health status, while the model’s predictions are plotted over time. These results suggest that the framework is effective for continuous health monitoring and could be integrated into precision agriculture decision-support systems.

### 4.3 Changes in plant health dynamics during the cultivation period

**Figure 11:**
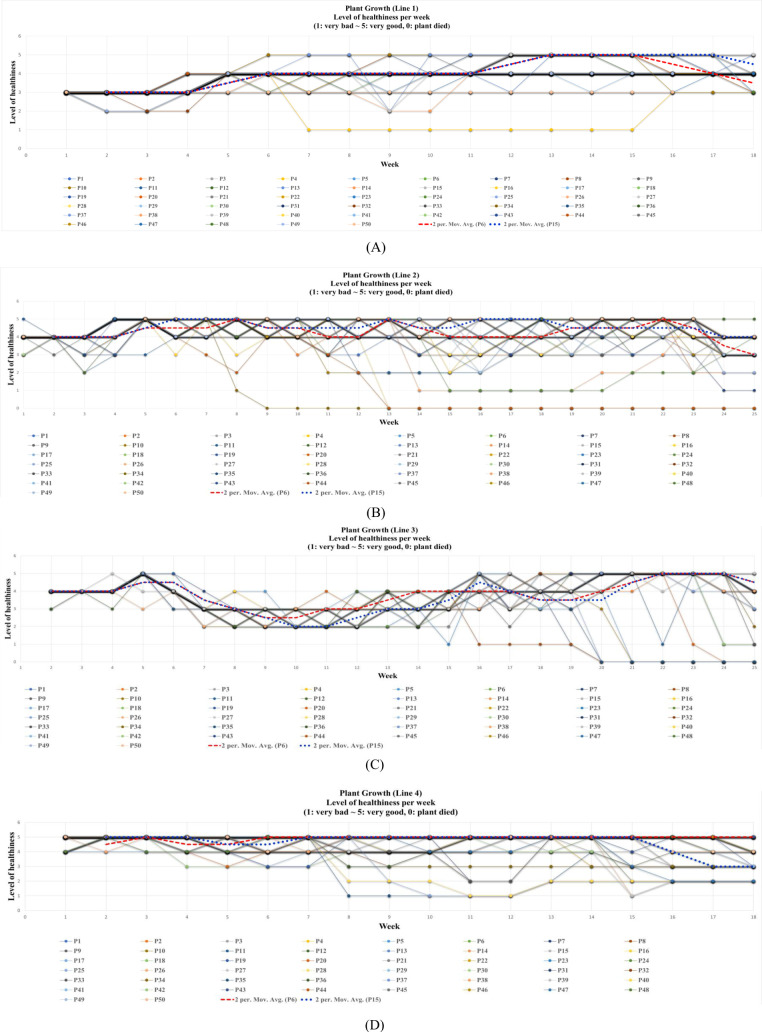
Temporal health status progression using expert annotations for: **(A)** Line 1 (Norari Cherry), **(B)** Line 2 (Amos Coli), **(C)** Line 3 (Amos Coli), and **(D)** Line 4 (Dafnis-Hybrid). P# represents the plant number per line. The dashed lines represent moving averages.

To analyze broader trends, **Figure 11** visualizes the evolution of plant health across all cultivation lines and plants using expert annotations. The horizontal axis represents time (weeks), while the vertical axis shows health states (1-5), including state 0 for plant mortality.

Each plotted point represents the health status of an individual plant in a given week, capturing fluctuations in health conditions. The red and blue dashed lines indicate moving averages, providing insight into overall trends. This visualization helps track:

- Health improvement or deterioration patterns across different plant varieties.
- The impact of external factors such as environmental stress or nutrient deficiencies.
- The potential for early intervention by identifying declining health states.
- Our findings suggest that early detection of declining plant health enables targeted interventions, improving overall crop resilience. This supports automated health monitoring as a valuable tool for precision agriculture.

### 4.4 Considerations for scalability, interoperability, and regulatory compliance

The successful deployment of the proposed plant health monitoring framework in real-world agricultural settings requires careful consideration of scalability, interoperability, and regulatory compliance. These factors determine the framework’s applicability to large-scale commercial farming, integration with existing agricultural technologies, and adherence to industry regulations, plant health monitoring systems must address three key criteria:

1. Scalability: For widespread adoption, the framework must efficiently scale across varied agricultural environments and handle large datasets with minimal computational overhead. Key aspects of scalability include:
  - Computational Efficiency: The deep learning models performing best in this study: Swin Transformer and ConvNeXt, require high computational resources. Deploying these models on edge devices or cloud-based infrastructures could facilitate real-time monitoring without reliance on centralized computing resources (O’Grady et al., 2019).
  - Extensibility to Other Crops: While this study focuses on tomato plants, the framework can be tested on different crop types to generalize beyond the specific datasets. However, collecting datasets from other crops may be also required.
  - Handling Large-Scale Deployments: As farms expand, the system must process thousands of images per day. Optimizations such as model distillation (Moslemi et al., 2024) could enhance efficiency, reducing inference time while maintaining accuracy.
2. Interoperability: For seamless integration into existing precision agriculture ecosystems, the framework must support interoperability with various data sources:
  - Multi-Modal Data Integration: Combining RGB image data with sensor readings (e.g., soil moisture, temperature, nutrient levels) can improve predictive accuracy (Talaviya et al., 2020). Future extensions should explore fusion models that integrate multi-modal data for crop monitoring.
3. Regulatory Compliance: Adhering to agricultural regulations ensures trust, security, and widespread adoption, including:
  - Data Privacy & Security: Given that plant health monitoring may involve farm-specific data, compliance with data protection.
  - AI Transparency & Accountability: Regulations such as the EU AI Act emphasize the need for explainable AI in critical applications, including agriculture (EU, 2024). The proposed Grad-CAM-based visualization contributes to transparency by enabling interpretable model decisions, which could be extended with model auditing frameworks to ensure fair and unbiased predictions.
  - Alignment with Agricultural Standards: The system should align with precision agriculture frameworks could enhance credibility among policymakers and agribusiness stakeholders (FAO, 2024).

## 5 Conclusion

This study proposed a deep learning-based framework for monitoring plant health throughout the entire cultivation period of tomato plants. Validated on four custom datasets representing different tomato varieties and growth stages, the framework achieved an outstanding performance, demonstrating its reliability for real-world applications. Key contributions include comprehensive dataset collection, enabling precise plant health assessments for early intervention, and showcasing scalability for precision agriculture. Despite these advancements, challenges such as class imbalance, generalization, and real-time deployment remain. Future work should explore multi-modal data integration, edge AI for real-time inference, and regulatory compliance to enhance adoption. The findings highlight the transformative role of deep learning in data-driven plant health monitoring, offering solutions to optimize crop management, sustainability, and farm productivity in modern agriculture.

## Data Availability

The datasets presented in this article are not readily available because the dataset is part of an ongoing project. Requests to access the datasets should be directed to afuentes@jbnu.ac.kr.
